# National Voice-Care Campaign and its importance in voice-care outreach activities

**DOI:** 10.1016/S1808-8694(15)30743-6

**Published:** 2015-10-19

**Authors:** Jeferson Sampaio D'Avila

**Affiliations:** Jeferson Sampaio D'Avila Head of Otorhinolaryngology - U.F.S. Chairman of the Brazilian Academy of Laryngology and Voice

I am pleased and honored to make some remarks about Laryngology in Brazil.

In recent decades, thanks to the hard work of our medical scientists, Brazil reached an unprecedented international prestige. This has also been true for laryngology. Initially, by developing and upholding a high internal standard, Brazilian laryngology not only was upgraded in relation to the rest of the world, but improved itself in such a way that it is today well regarded and respected in all continents, thanks to the relevance of our research as well as the excellent representativeness from our team of laryngologists.

In these specific grounds, our Brazilian Academy of Laryngology and Voice (ABLV) carried a large responsibility. Always very well chaired along all these years, the academy has been able to convey a high level of credibility both in the country and abroad, which created this elevated social and medical acknowledgement.

Starting from this base, and being an integral part of this development, the Voice-Care Campaign started 10 years ago. Based on information disclosure, the campaign has been able to alert the general population about the causes of laryngeal diseases, how to avoid and treat them, providing the Brazilian population a deeper awareness on the topic. Ultimately, this resulted not only in a greater number of visits to specialized services, but also in earlier diagnoses of throat diseases, especially those associated with the vocal folds. This campaign also served to bring Brazilians a greater awareness of Otorhinolaryngology. In order to toast to this excellent ABLV work, the World Voice-Care Day was created, which is internationally held every April 16.

In our present administration the Voice-Care Campaign has finally become permanent, a fact that celebrates the 10th Campaign and the 6th World Voice-Care Day. With this action, the disclosure results became more evident and we believe that at the 2009 World Convention, Brazilian representativeness in the world, through the ABLV, will represent a historical landmark for Brazilian Otorhinolaryngology.

